# Cancer Preventive and Therapeutic Potential of Banana and Its Bioactive Constituents: A Systematic, Comprehensive, and Mechanistic Review

**DOI:** 10.3389/fonc.2021.697143

**Published:** 2021-07-07

**Authors:** Arijit Mondal, Sabyasachi Banerjee, Sankhadip Bose, Partha Pratim Das, Elise N. Sandberg, Atanas G. Atanasov, Anupam Bishayee

**Affiliations:** ^1^ Department of Pharmaceutical Chemistry, Bengal College of Pharmaceutical Technology, Dubrajpur, India; ^2^ Department of Pharmaceutical Chemistry, Gupta College of Technological Sciences, Asansol, India; ^3^ Department of Pharmacognosy, Bengal School of Technology, Chuchura, India; ^4^ Lake Erie College of Osteopathic Medicine, Bradenton, FL, United States; ^5^ Ludwig Boltzmann Institute for Digital Health and Patient Safety, Medical University of Vienna, Vienna, Austria; ^6^ Institute of Genetics and Animal Biotechnology of the Polish Academy of Sciences, Magdalenka, Poland; ^7^ Department of Pharmacognosy, University of Vienna, Vienna, Austria

**Keywords:** cancer, banana, *Musa accuminata* Colla, *Musa balbisiana* Colla, *Musa* spp., prevention, therapy, molecular mechanisms

## Abstract

**Background:**

The banana (*Musa* spp.) plant produces elongated and edible fruit. The two main parthenocarpic species of banana are *Musa accuminata* Colla and *Musa balbisiana* Colla. There are several health-promoting and disease-preventing effects of *Musa accuminata* Colla, which are attributed to its important bioactive compounds, including phenolics, carotenoids, biogenic amines, phytosterols, and volatile oils, found in the stem, fruit, pseudostem, leaf, flower, sap, inner trunk, root, and inner core. Banana possesses numerous pharmacological activities, such as antioxidant, immunomodulatory, antimicrobial, antiulcerogenic, hypolipidemic, hypoglycemic, leishmanicidal, anthelmintic, and anticancer properties. Various individual studies have reported anticancer effects of different components of the banana plant. However, according to our understanding, an up-to-date, systematic, and critical analysis of existing scientific results has not yet been carried out.

**Objectives:**

This review aims to include a thorough assessment of banana and its phytochemicals for cancer prevention and therapy with a focus on cellular and molecular mechanisms of action.

**Methods:**

The available research studies on anticancer activities of banana extracts, fractions and pure compounds were collected using various scholarly databases, such as PubMed, ScienceDirect, and Scopus, based on predetermined selection criteria.

**Results:**

Various banana extracts, fractions, and phytoconstituents, including ferulic acid, protocatechualdehyde, 2-pentanone, 4-epicyclomusalenone, cycloeucalenol acetate, and chlorogenic acid, have been shown to exhibit cancer preventative and anticancer activities in breast, cervical, colorectal, esophageal, hepatic, oral, prostate, and skin cancers. Bioactive components present in bananas have exhibited antiproliferative, cell cycle arrest-inducing, apoptotic, anti-adhesive, anti-invasive, and antiangiogenic effects through modulation of diverse, dysregulated oncogenic signaling pathways.

**Conclusion:**

Based on the critical analysis of available literature, banana products and phytoconstituents show enormous potential for future development of drugs for cancer prevention and therapy. However, more mechanistic studies and well-designed clinical trials should be performed to establish its efficacy.

## Introduction

Cancer, the second most frequent cause of mortality, is a hyper-proliferative disorder that involves cellular transformation, deregulation of apoptosis, and excessive proliferation, invasion, angiogenesis, and metastasis (1). Despite innovative therapeutic approaches and newer technological developments, cancer continues to be amongst the most fatal disorders (2, 3). According to the 2020 Global Cancer Observatory report provided by the International Agency for Research on Cancer (World Health Organization), there were 18.1 million new cases of cancer and 9.5 million cancer-related deaths that occurred globally in 2018. They also stated that the number of newly diagnosed cancer cases is projected to increase to 29.5 million per year, and projected cancer-related deaths are expected to increase to16.4 million per year by 2040 (www.cancer.gov). While the precise cause for cancer initiation is still unclear, the most important contributing variables for this condition are toxins, pollution, radioactive substances, oncogenic viruses (4, 5), and epigenetic abnormalities (6). Many anticancer medications currently in use not only destroy cancer cells, but also healthy cells too. The major obstacles involved with cancer chemotherapy are non-specific targeting and the evolution of drug resistance. The need for more effective anticancer medications with improved safety profiles has become an urgent need to defeat this dreaded disease, despite significant advances in cancer screening, diagnosis, and treatment.

For most of the world, modern medicine has replaced traditional medicine as means of therapy for human illnesses (7). Nevertheless, the use of medicinal plants for health promotion and disease prevention has increased in recent decades (8). Phytochemicals are being extensively investigated, and they have demonstrated promising anticarcinogenic properties by interfering with cancer initiation and modulating various pathways, including cell proliferation, differentiation, apoptosis, angiogenesis, invasion, and metastasis (9–12).

The term “banana” refers to the cultivated varieties of the genus *Musa*, which are made up of two subgroups: sweet bananas and plantains (13). *Musa*, *Ensete*, and *Musella* are the three genera of the Musaceae family (14), with the *Musa* genus comprising of 65 species of wild and cultivated bananas and plantains. Banana has been described in ancient Indian treatises, including the Ramayana (2000 BC), Arthsastra (250 BC), and Chilappthikaram (500 AD), suggesting the fruit’s importance and demonstrating its ancient use in India. The genus name *Musa* was chosen to commemorate Roman physician and botanist Antonius Musa (63 BC-14 AD) (15). Historically, *Musa acuminata* has been discovered in the native habitats of India (15). At present, banana is cultivated around the world, and the significant producers of banana fruits are India (29 million tonnes/year), China (11 million tonnes/year), Philippines (7.5 million tonnes/year), and Brazil, as well as Ecuador, which produces 7 million tonnes/year on average (16).

**Figure 1:**
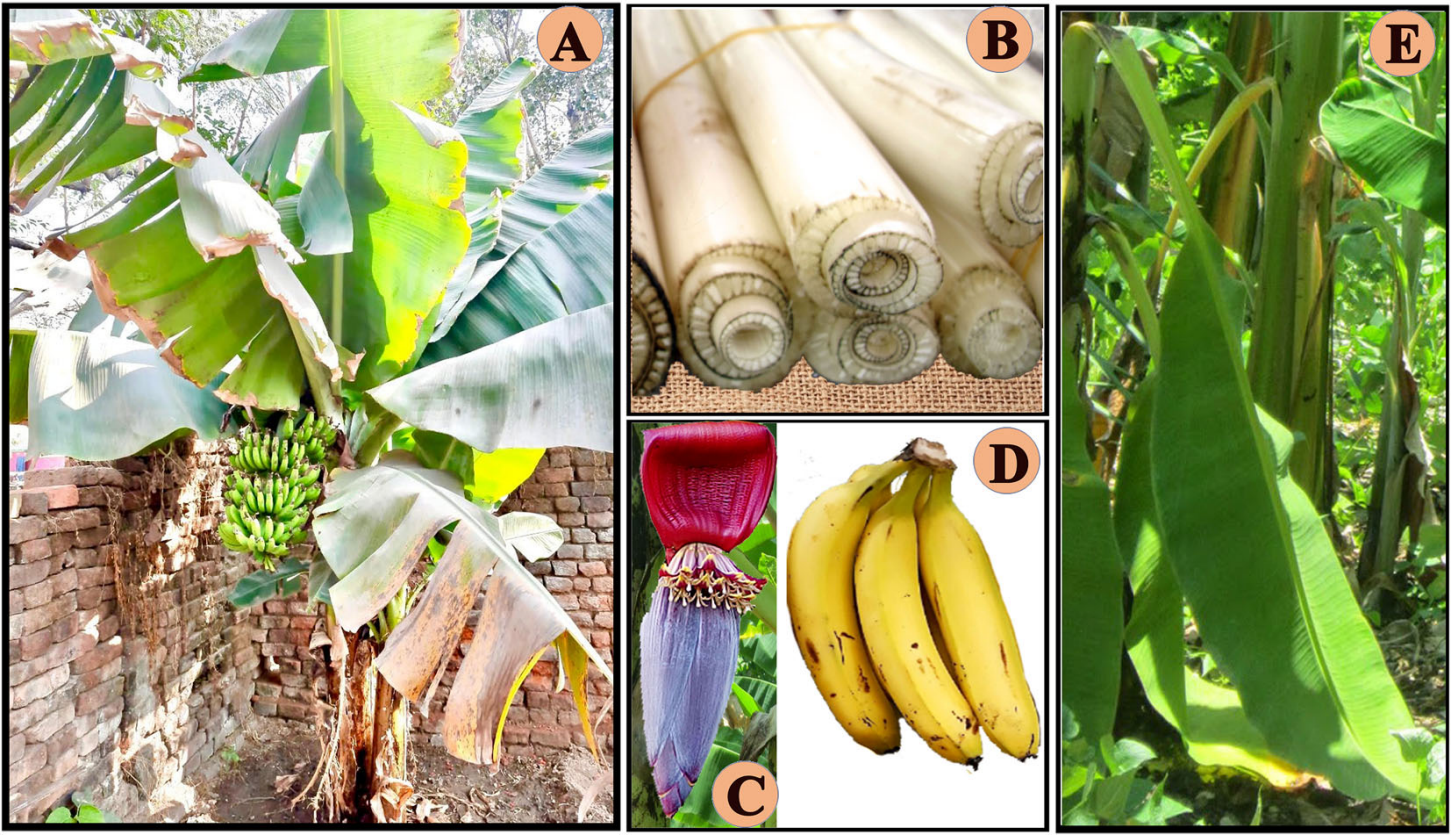
Various photographs of banana showing a whole plant **(A)**, pseudostem **(B)**, flowers **(C)**, fruits **(D)** and leaves **(E)**.

The banana is a perennial herb that looks like a tree (**Figure 1A**). It forms shoots that emerge from the rhizome’s lateral buds, which then grow into fruit-bearing stems. A pseudostem (**Figure 1B**) begins to appear like a trunk, however it is actually is a compact assembly of wrapping, spirally arranged leaf sheaths. The banana plant’s flowers (**Figure 1C**) produce a big spike, which subsequently opens by turning downward towards the soil. A single plant produces both male and female flowers. The fruits (**Figure 1D**) are green or yellow in color, have a long shape, and are produced in bunches and clusters. Each leaf (**Figure 1E**) arises from the pseudostem’s center. The elongating leaf sheath’s distal end expands into a petiole. The midrib, which splits the blade into two lamina halves, is formed by the petiole (17).

Nearly every portion of the banana plant has its essential use and is beneficial in many respects to mankind. Traditionally, *M. acuminate* plants have been used to treat non-communicable as well as transmissible diseases, especially in Asia and Africa. It has been extensively used by indigenous people as both food and medicine (18, 19). Various parts of the plant, such as the stem, fruit, pseudostem, leaf, vine, sap, inner trunk, root, and inner core, have been used in the management of various diseases, including the regulation of blood pressure (20), diabetes (18), hypertension (21), anemia (22), allergic reaction, microbial infections, and chronic bronchitis disorders. *M. acuminate* has also been used for the treatment of fever, cough, tuberculosis, and dysentery by many tribes and communities (23, 24). The root extract has been utilized to prevent conception (25), stimulate labor (26), and cure infections of sexually transmitted diseases, such as human immunodeficiency virus-related infections, internal and external sores of the genitalia, vaginitis and leucorrhoea (27, 28). Some common uses recorded in the literature include its usefulness as an anthelmintic, as dressing for cuts and blistered skin, and as a liqueur to reduce joint inflammation and promote blood circulation (15). Aside from the conventional uses mentioned earlier, the *Musa* species has also been documented to possess pharmacological activities, demonstrating antioxidant, immunomodulatory, antimicrobial, anticancer, antiulcerogenic, hypolipidemic, hypoglycemic, leishmanicidal, and anthelmintic properties (29).

Many bioactive phytoconstituents of bananas have been isolated, characterized, and analyzed for their anticancer properties; however, no previous reviews offer a systematic analysis of published anticancer studies of *Musa* species. There are only a limited number of prior publications that provide a brief overview of the nutritional values of banana and its overall pharmacological activities (29, 30). There are no articles focusing on the antineoplastic properties of *Musa* species, their phytoconstituents in different kind of cancers and related signaling pathways. While there are numerous and emerging information on anticancer properties of banana and its constituents based on laboratory and clinical findings, a comprehensive assessment of cancer preventive and therapeutic characteristics of banana, banana products, extracts, fractions, and isolated phytochemicals has yet to be performed. In view of this limitation, our current work elucidates cancer preventive and therapeutic potential of banana and its bioactive phytoconstituents observed in several types of cancers and highlights underlying mechanisms of action, which involve targeting various cell signaling pathways and molecules.

## Phytochemical Profiles of *Musa* Species

The phytochemical analysis of different anatomical parts of banana plant, such as the leaves, fruits, peels, flowers, pseudostems, and rhizomes, has shown that there are numerous secondary plant metabolites, including polyphenols, terpenoids, alkaloids, steroids, anthocyanins, tannins, and fatty acids (**Table 1**). Banana fruit has been recorded as a significant source of phenolic compounds, including phenolic acids, flavonoids, and glycosides. The pulps and peels of banana and plantain have demonstrated potential to be utilized by the pharmaceutical and food industries for their catechin and rutin content (60, 61). Plantain pulps and peels are also a good resource of phenolic compounds. Recent examinations of plantains have revealed that hydroxycinnamic acids represent the majority of phenolic compounds in the fruit pulp, while flavonoids are mostly found in higher concentrations in the peel (62).

**Table 1:** Phytochemicals identified in various parts of *Musa* spp.

Plant parts	Category	Phytochemicals from various banana cultivars	Quantitative values	Extract	References
Ripe fruit	Phenolic acids	Octadeca-9,12,15 trienoic acid (**1**)	36-405 mg/kg of dry material	Dichloromethane	(31)
		Octadeca-9,12-dienoicacid (**2**)	12-198 mg/kg of dry material		
		Vanillic acid (**3**)	8.54 mg/100g of acetone extract	Acetone	(32)
		Caffeic acid (**4**)	1.36 mg/100g of acetone extract		
		Ellagic acid (**5**)	68.82 mg/100g of acetone extract		
Peel		13-octadecanoic acid (**6**)	5.59%	Methanol, oil	(29, 33–35)
		Palmitic acid (**7**)	30%		
		Oleic acid (**8**)	7%		
		Linoleic acid (**9**)	8%		
		Methyl palmitate (**10**)	–		
		Methyl oleate (**11**)	–		
		Methyl linoleate (**12**)	–		
		Stearic acid (**13**)	2%		
		Carvacrol (**14**)	–		
		Pentadecanoic acid (**15**)	18.81% of extract		
		Palmitoleic acid (**16**)	–		
		Benzoic acid (**17**)	16.04% of extract		
Leaves		Tannic acid (**18**)	7.04-12.19 mg/ml	Ethanol, acetone, petroleum ether	(36)
		Cinnamic acid (**19**)	43-80 ng/g dry weight	Leaf powder	(37)
		Ferulic acid (**20**)	2680-5900 ng/g dry weight		
Bract		Delphinidin-3-rutinoside (**21**)	0.00-66.70 mg/100 g	Methanol	(38–40)
		Cyanidin-3-rutinoside (**22**)	0.00-37.52 mg/100 g		
		Petunidin-3-rutinoside (**23**)	0.00-11.91 mg/100 g		
		Peonidin-3-rutinoside (**24**)	0.00-36.92 mg/100 g		
		Malvidin-3-rutinoside (**25**)	0.00-70.27 mg/100 g		
Seeds		Leucoanthocyanidin (**26**)	–	Acetone	(29)
Pulp of banana fruit		Gallic acid (**27**)	–	Not specified	(41)
Over ripe fruit		Protocatechualdehyde (**28**)	–	Not specified	(42)
Sap		Hydroxycinnamic acid (**29**)	24-45% of sap	Ethanol	(43)
		Caffeoylquinic acid (**30**)	24-45% of sap		
Ripe fruit	Flavonoids	Quercetin (**31**)	6.5-18.9 µg/100 g of dry weight pulp	Pulp	(41, 44)
		Proanthocyanidin (**32**)	–		
		Catechin (**33**)	33.3-143.2 µg/100 g of dry weight pulp		
		Gallocatechin (**34**)	37.3-542 µg/100 g of dry weight pulp	Methanol	(41, 45)
		Epicatechin (**35**)	17.9-459.8 µg/100 g of dry weight pulp		
		Procyanidin (**36**)	1.6-124.7 µg/100 g of dry weight pulp		
		(+) Catechin hydrate (**37**)	23.34 mg/100 g of acetone extract	Acetone	(32)
Sap		Apigenin (**38**)	5.50-23.81% of sap	Ethanol	(43)
		Myricetin (**39**)	1-45% of sap		
		Kaempferol (**40**)	2.89-23.50% of sap		
Ripe fruits	Glycosides	Endo-*β*-1,3-glucanase (Ban-Glu) (**41**)	208 and 237 amino acids are present in two varieties	Pulp	(46)
		*α*-tocopherol (**42**)	2-7 mg/kg of dry material	Dichloromethane	(31)
	Triterpenoids	Cycloartenol (**43**)	1-4 mg/kg of dry material	Dichloromethane	
	Sterols	Campesterol (**44**)	18-59 mg/kg of dry material	Dichloromethane	
		Stigmasterol (**45**)	23-49 mg/kg of dry material		
		*β*-sitosterol (**46**)	105-226 mg/kg of dry material		
Peels		Pyrogallol (**47**)	22.24%	Methanol	(33)
Ripe banana		Sitosteryl glucoside (Sitogluside) (**48**)	–	Not specified	(47)
Peel	Lignan	Sesamin (**49**)	–	Methanol, oil	(48)
Peel		Epi-sesamin (**50**)	–		
Ripe fruit	Carotenoids	Ascorbic acid (**51**)	–	Pulp, Peel	(29)
		Retinol (**52**)	–		(49)
		*α*-carotene (**53**)	–		(50, 51)
		*β*-carotene (**54**)	–		
		Zeaxanthin (**55**)	–		
Unripe fruit	Miscellaneous	2-(4-hydroxyphenyl)-naphthalic anhydride (**56**)	–	Pulp	(52)
		methyl 2-benzimidazole carbamate (**57**)	–		
Peel		Dopamine (**58**)	3.9-381 mg/100 dry weight banana peel extract	Acetone:water	(53)
Ripe fruit		Serotonin (**59**)	1-2 mg/100 g	Not specified	(54, 55)
		Histamine (**60**)	0.04 mg/100 g		
		Tryptamine (**61**)	0.06 mg/100 g		
		2-phenylethylamine (**62**)	0.04 mg/100 g		
		Putrescine (**63**)	0.04 mg/100 g		
		Cadaverine (**64**)	0.04 mg/100 g		
		Tyramine (**65**)	0.06 mg/100 g		
Peels		2,3-dihydro-3,5-dihydroxy-6-methyl-4H-pyran-4-one (**66**)	–	Methanol, oil	(29, 33, 48)
		5-(hydroxymethyl)-2- Furancarboxyaldehyde (**67**)	–		
		cis-9-hexadecenal (**68**)	21.20%		
Rhizome		(S)(+)-naproxene (**69**)	–		(56–58)
		2-methoxy-9-phenyl-phenalen-1-one (**70**)	0.3 mg/478 mg of peel extract		
Rhizome, Root		Anigorufone (**71**)	–	Methanol	(29)
Fruit		2-pentanone (**72**)	4.8-6.0 mg/kg	Not specified	(59)
Sap		N-acetylserotonin (**73**)	17-34.76% of sap	Ethanol	(43)

Borges et al. (60) proposed that parental combinations of banana genotypes of *M. acuminata *and *M. balbisiana* may be chosen for hybrid production. The biofortification of *Musa* spp. produced diploid, triploid, and tetraploid hybrids which contained higher amounts of bioactive compounds than non-hybrid plants. Several studies have reported catechin, epicatechin, and gallocatechin as major compounds in the triploids cultivars Highgate (AAA) genotype of *M. acuminata *and *M. balbisiana* which have an epicatechin content of 114.44 mg/100 g dry weight basis and gallocatechin content of 591.41 mg/100 g dry weight basis. In addition to catechin compounds, protocatechuic acid, gallic acid, 7-*O*-neohesperoside naringenin, and hydroxycinnamic acids have additionally been identified in banana pulp (41, 60, 62, 63).

**Figure 2:**
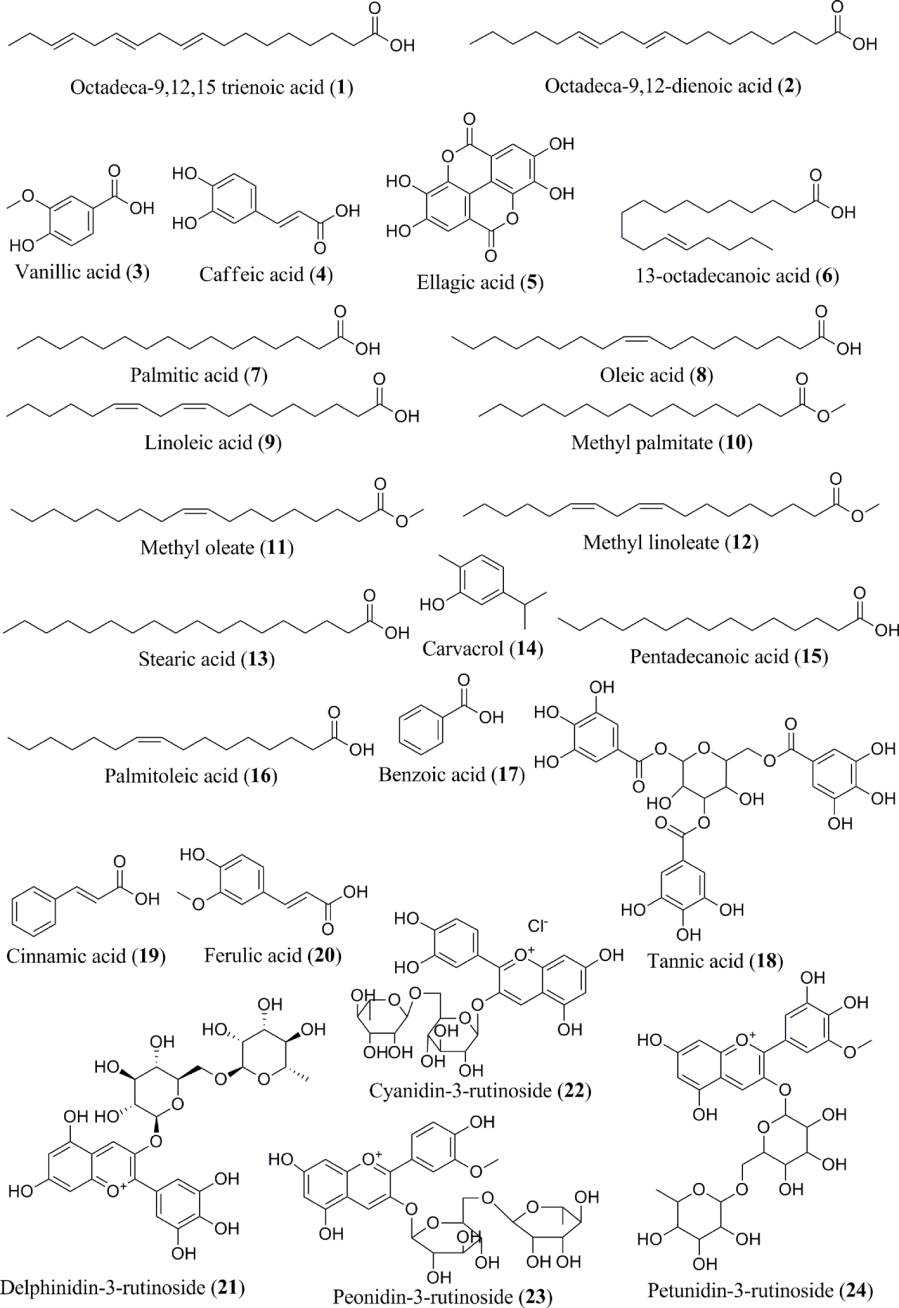
Isolated phytoconstituents (**1–24**) present in *Musa* spp.

**Figure 3:**
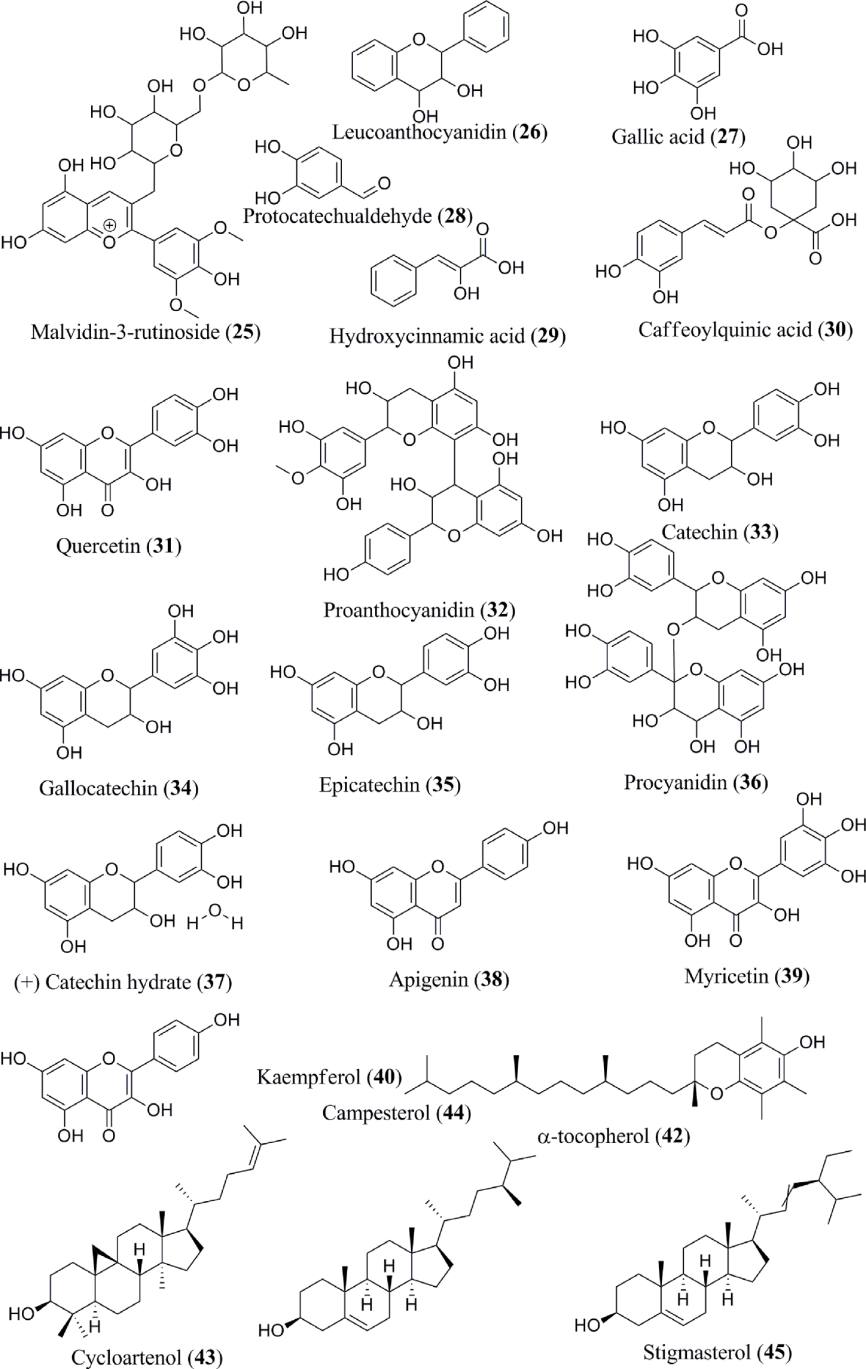
Isolated phytoconstituents (**25–45**) present in *Musa* spp.

The content of phenolic compounds varies in the raw and ripe fruits. Thermal treatments of the banana fruits weakened the cell wall and facilitated the release of phenolic component, such as ferulic acid (62). Boiled plantain pulps with or without peel demonstrated an increased number of phenols in pulps (62). Additionally, protocatechualdehyde, a naturally occurring polyphenol, was isolated, purified, and characterized in green cavendish bananas (42). The phenolic compounds collectively present in the banana are octadeca-9,12,15 trienoic acid (**1**) (**Figure 2**), octadeca-9,12-dienoicacid (**2**), vanillic acid (**3**), caffeic acid (**4**), ellagic acid (**5**), 13-octadecanoic acid (**6**), palmitic acid (**7**), oleic acid (**8**), linoleic acid (**9**), methyl palmitate (**10**), methyl oleate (**11**), methyl linoleate (**12**), stearic acid (**13**), carvacrol (**14**), pentadecanoic acid (**15**), palmitoleic acid (**16**), benzoic acid (**17**), tannic acid (**18**), cinnamic acid (**19**), ferulic acid (**20**), delphinidin-3-rutinoside (**21**), cyanidin-3-rutinoside (**22**), petunidin-3-rutinoside (**23**), peonidin-3-rutinoside (**24**), malvidin-3-rutinoside (**25**) (**Figure 3**), leucoanthocyanidin (**26**), gallic acid (**27**), protocatechualdehyde (**28**), hydroxycinnamic acid (**29**), caffeoylquinic acid (**30**), quercetin (**31**), proanthocyanidin (**32**), catechin (**33**), gallocatechin (**34**), epicatechin (**35**), procyanidin (**36**), (+) catechin hydrate (**37**), apigenin (**38**), myricetin (**39**), kaempferol (**40**), endo-β-1,3-glucanase (Ban-Glu) (**41**), and α-tocopherol (**42**).A triterpenoid, namely cycloartenol (**43**), was extracted from the dichloromethane extract of ripe pulp of various banana cultivars (29, 31–41, 43–46).

**Figure 4:**
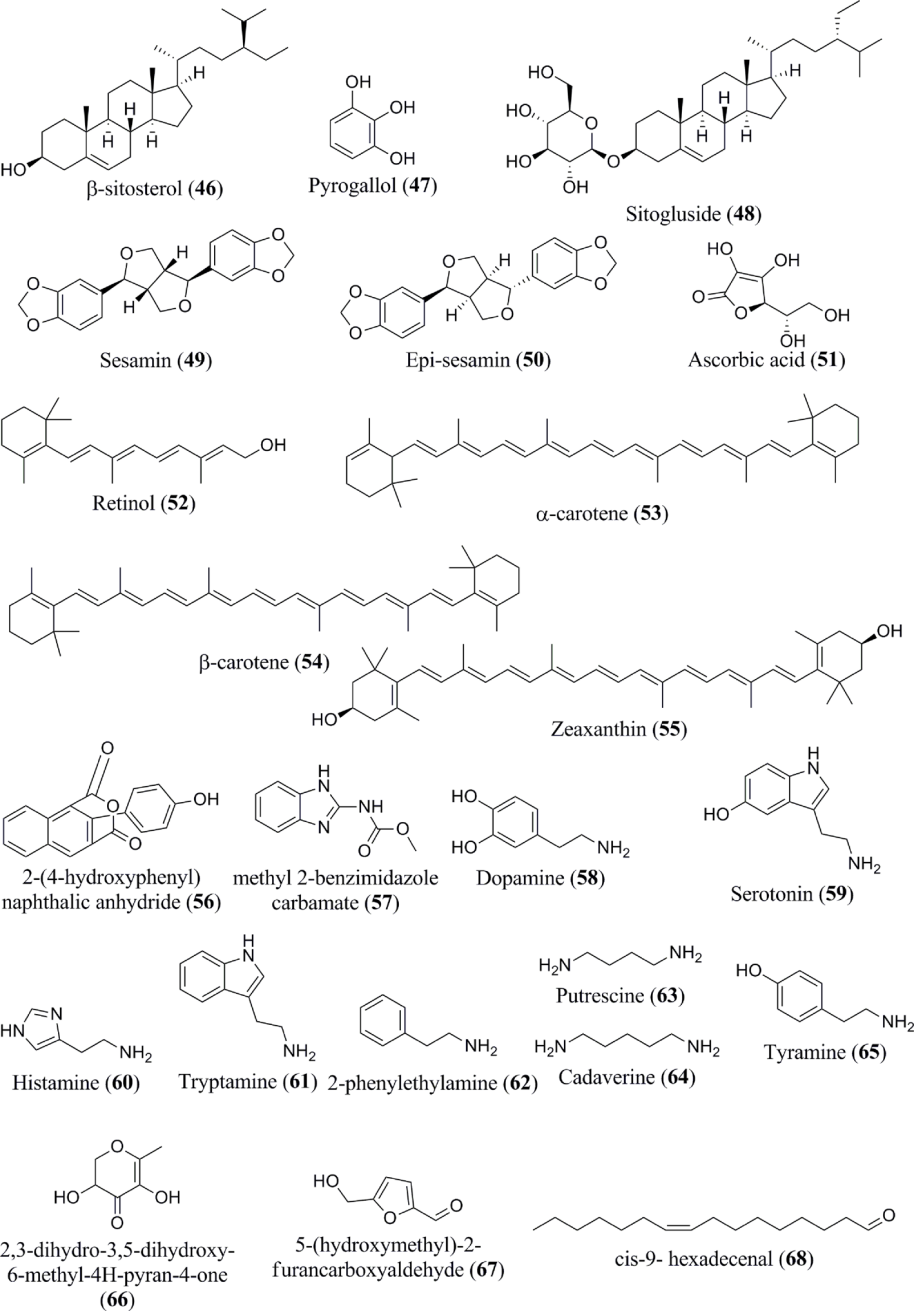
Isolated phytoconstituents (**46–68**) present in *Musa* spp.

Banana fruits have substantial phytosterol concentrations (64). Several sterol components are present in the *Musa* spp., namely, campesterol (**44**), stigmasterol (**45**), β-sitosterol (**46**) (**Figure 4**), which exist in the dichloromethane extract (31). The methanol extract of banana peel contains pyrogallol/benzene-1,2,3-triol (**47**) (33). In addition to these phytochemicals, sitosteryl glucoside (sitogluside) (**48**) is present in ripe banana fruits (47). Two kinds of lignan, namely sesamin (**49**) and epi-sesamin (**50**), have also been isolated and identified in the methanol extract of banana peel (48).

Carotenoids, vitamin C (ascorbic acid) (**51**), and vitamin A (retinol) (**52**) are the most abundant antioxidants present in the banana pulp and peel (49). Significant quantities of bioactive carotenoids have been extracted and identified in *Musa* spp. biomasses as well (50, 51). Various banana genotypes may even produce higher amounts (approx. 90%) of vitamin A precursors, such as α- and β-carotene (**53** and **54**) (65). Zeaxanthin (**55**) has additionally been found in bananas. Several additional novel compounds, such as 2-(4-hydroxyphenyl)-naphthalic anhydride (**56**), and methyl 2-benzimidazolecarbamate (**57**), were reported to be present in banana (52).

**Figure 5:**
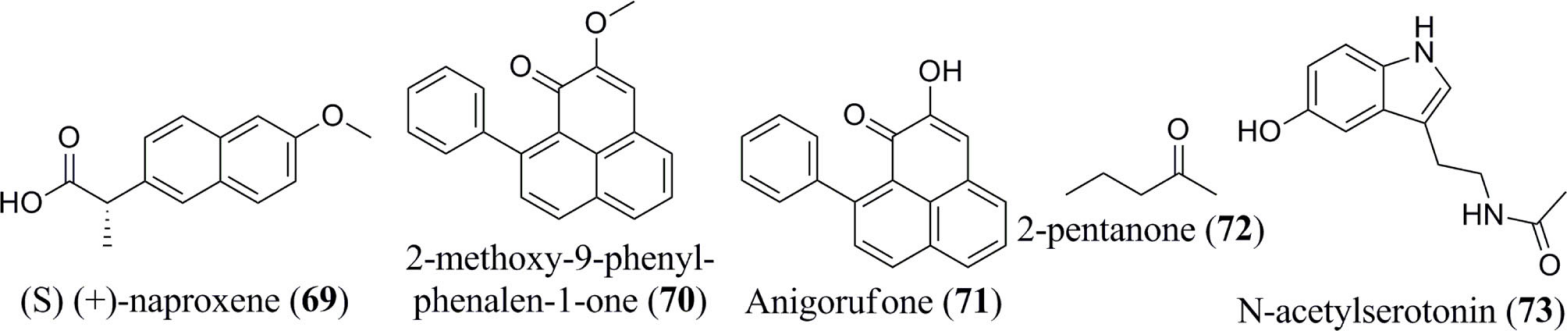
Isolated phytoconstituents (**69-73**) present in *Musa* spp.

According to Xiao et al. (66), the consumption of banana, which is considered relatively rich in serotonin, leads to a rapid elevation of the hormone level within the blood. The quantity and specific type of biogenic amines formed are affected by the composition of the plant’s nutritional intake, microbial flora, storage (e.g., degree of ripeness and temperature), and processing to which they are subjected (54, 67, 68). The most common amine compounds are spermidine and spermine. Diamines, such as putrescina and agmatina, are precursors of these polyamines (69). Dopamine (**58**), serotonin (**59**) and histamine (**60**) have all been detected in bananas and their by-products (54). Serotonin has been extracted in higher amounts in the fruits of *Musa* spp., particularly when compared with other fruits and vegetables (32). Additionally, several biogenic amines, tryptamine (**61**), 2-phenylethylamine (**62**), putrescine (**63**), cadaverine (**64**), and tyramine (**65**), are also present in banana (55). Interestingly, a ketone compound, namely 2-pentanone, has been isolated from bananas as well (59). Several other miscellaneous components are reported to exist in *Musa* spp., including 2,3-dihydro-3,5-dihydroxy-6-methyl-4H-pyran-4-one (**66**), 5-(hydroxymethyl)-2-furancarboxyaldehyde (**67**), cis-9- hexadecenal (**68**), (S) (+)-naproxene (**69**) (**Figure 5**), 2-methoxy-9-phenyl-phenalen-1-one (**70**), Anigorufone (**71**), 2-pentanone (**72**), and N-acetylserotonin (**73**) (33, 43, 48, 56–59). The phytoconstituent compositions of different banana species (**Table 1**) differ quantitatively due to soil, temperature, banana type, maturation stage, processing site, and other factors (70).

## Safety Profile of Banana

Local and tribal communities have discovered that the use of banana fruit and other parts of the banana plant is non-toxic. Banana fruits and other plant parts are consumed by indigenous populations throughout the world. The banana (*M. acuminata*) flower is used to prepare a popular Sri Lankan dish as a curry, boiled or deep-fried salad (71). There were no adverse consequences upon the administration of banana (*M. acuminata*) extracts in preclinical trials (22, 72–74). The flowering stalk of *M. acuminata* was documented to be non-toxic against the murine monocytic macrophages cell line (75). A toxicity examination in brine shrimp (administered in *Artemia salina*) revealed that *M. acuminata* flower extract was safe as well (76). Likewise, the utilization of *M. acuminata* peel as an ingredient in food products suggests that it’s considered safe for consumption (77–79). Furthermore, banana peel exhibited no toxicity towards normal human cells (80). According to acute and subacute toxicity analysis, aqueous fermented extract of *M*. *paradisiaca *plantain was reported to be non-toxic and considered safe when administered to rats at a dose of 800 mg/kg body weight, which revealed no significant changes in the hematological and serum biochemical parameters or histopathological studies of the liver and kidney (81). According to Abbas et al. (82), the methanol extract of *M*. *paradisiaca* (bract and flowering stalk) showed potent nephroprotective activity in gentamicin-induced nephrotoxicity in mice. Cellulose nanofibers isolated from the banana peel (*M*. *paradisiaca*) exhibited no cytotoxicity against Caco-2 cell line (83). The methanol extract of *M. paradisiaca* root exhibited no signs of toxicity or mortality in broiler chickens with doses up to 4000 mg/kg body weight (84). Similarly, the hydro-ethanol extract of pseudostem of *M. paradisiaca* did not demonstrate any signs of toxicity or mortality in male Wistar rats when administered doses of up to 3000 mg/kg body weight (85). Additionally, the hydro-methanolic extract of *M. balbisiana* flower exhibited its non-toxic effects in streptozotocin-induced diabetic male albino Wistar rats (86). Aqueous extract of fresh ripe peel (*M. sapientum* Linn.) also did not induce any cytotoxicity in RAW 264.7 murine macrophage cell lines, which was evident from the presence of 70% viable cells (87).

## Cancer Preventive And Anticancer Therapeutic Potential of *Musa Spp*.

### Literature Search Methodology

**Figure 6:**
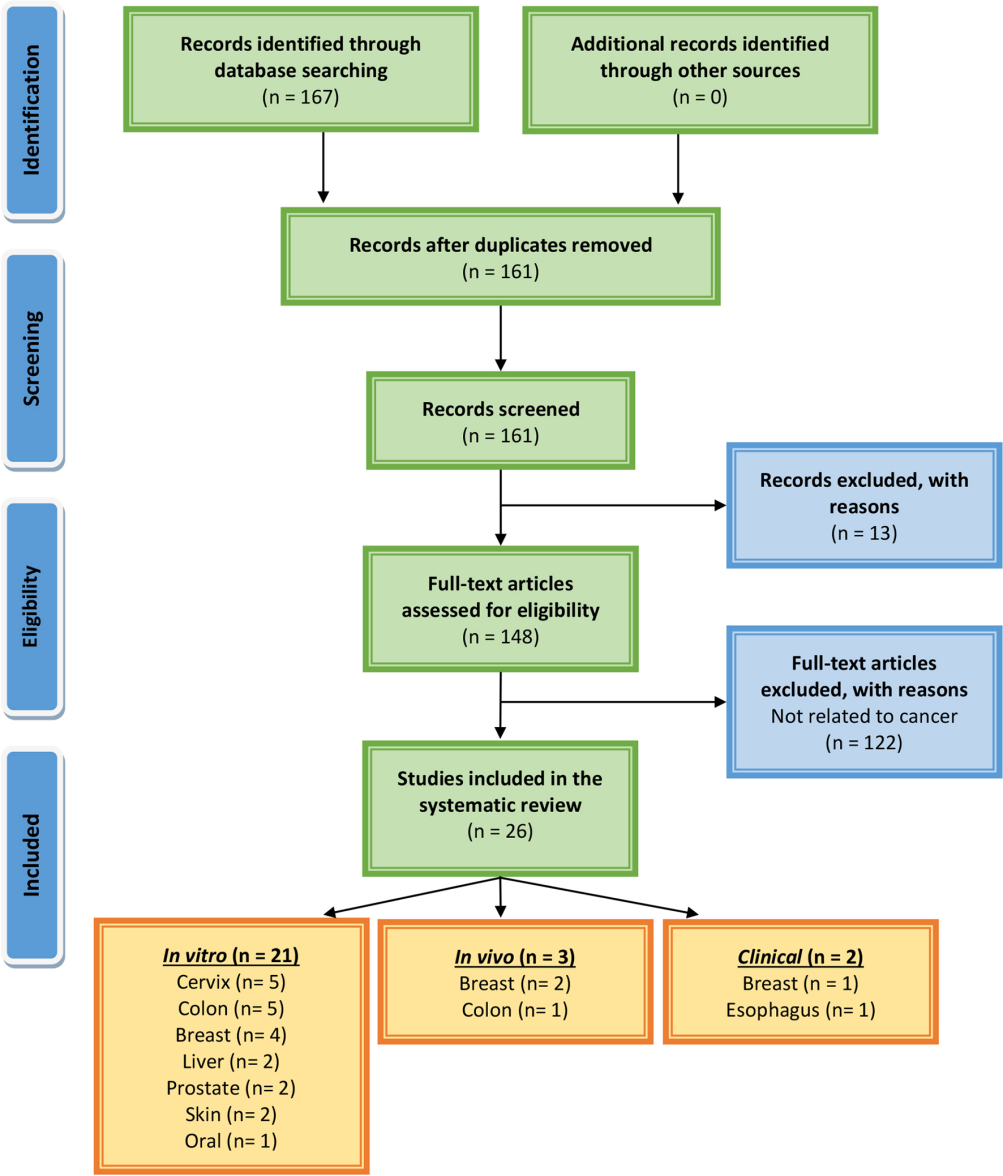
PRISMA flow chart describing the process of literature search and study selection related to banana in cancer research.

We have followed the guidelines of Preferred Reporting Items for Systemic Reviews and Meta-Analysis (PRISMA) (88) which is a credible process utilized for systematic analysis compilation (**Figure 6**). The major databases used to find primary literature were PubMed, ScienceDirect, and Scopus. Additionally, clinical trials were searched using clinicaltrials.gov. There were no time restraints on research articles that were published. The last search was performed in March 2021. Various combinations of keywords that were used included: *Musa* species; banana; chemopreventive, chemotherapeutic, *in vivo*, *in vitro*, cancer, tumor, prevention, treatment, proliferation, apoptosis, and clinical studies. We only considered studies that investigated anticancer effects on banana extracts or constituents against cancer cell lines and/or animal tumor models. Initially, the abstracts of all publications were reviewed to determine the next step, i.e., the collection of full-length articles. Once a full article was reviewed, a decision was made regarding its incorporation for further analysis. Only reports published in the English language were included. Reviews, systemic reviews, meta-analyses, letters to editors, book chapters, and conference abstracts were excluded. These searches were also performed by reviewing the bibliography sections of published papers.

### Preclinical Studies

Bananas showed a predominance of flavonoids, cinnamic acids, and polyphenolic compounds, which exhibit chemopreventive potential through various pathways analysed through *in vitro* (**Table 2**) and *in vivo* (**Table 3**) studies. Banana, as well their bioactive compounds, which exhibited anticancer, cytotoxic, and antiproliferative activity against various cancer types, are discussed below.

**Table 2:** *In vitro* anticancer activities of *Musa* sp., extract and its phytoconstituents.

Materials tested	Cell line used	Effects and mechanisms	EC_50_/IC_50_	References
*Breast cancer*				
Aqueous methanol extract of *Nendran* banana peel	MCF-7 breast cancer	↓Cell viability; ↑apoptosis	120.6 μg/mL	(89)
Methanol extracts from *Musa acuminata* bract		┴Proliferation; ↑apoptosis	12.24% inhibition at 1000 μg/mL	(90)
*Musa cavendish* green peel hydroaloholic extract		┴Proliferation	100 μg/mL	(91)
Hexane extract of banana (*Musa sapientum*) peel and pulp		↑Cytotoxicity; ┴proliferation	48.22% inhibition at 50 µg/mL (peel) and 61.21% inhibition at 50 μg/mL (pulp)	(92)
*Cervical cancer*				
Ethanol extract of banana flower	HeLa cells	↑Cytotoxicity; ┴proliferation; ↑apoptosis; ┴cell cycle at G_0_/G_1_ phase; ↑caspase-9 activity	20 μg/mL	(93)
Mannose specific *Musa acuminata* Lectin (MAL) from the phloem exudates of *M. acuminata* pseudostem	HeLa cells	↑Cytotoxicity; ┴proliferation; ↑apoptosis; ↑DNA fragmentation; ┴cell cycle at G_2_/M phase; ↓Bcl-2; ↑Bax; activation of caspase-3, caspase-8 and caspase-9; ↑cleaved PARP; ┴pAkt; ┴p-ERK1/2; ┴p-JNK	13.25 μg/mL	(94)
Ferulic acid from banana peel using *Staphylococcus aureus*	HeLa cells	↓Cell viability; ↑cytotoxicity; ↑DNA fragmentation	125μg/mL	(95)
Methanolic flower extract of *Musa acuminata*	HeLa cells	↑Cytotoxicity; ┴proliferation	71.9% cytotoxicity at 100 μL	(96)
*Musa Paradisiaca* L. leaves ethyl extract	HeLa and A375 cell lines	↑Cytotoxicity	249.1 and 224.4 μg/mL	(97)
*Colon cancer*				
Hexane extract of banana (*Musa sapientum*) peel and pulp	HCT-116	↑Cytotoxicity	62.04% inhibition at 50 µg/mL (peel) and 32.76% inhibition at 50 μg/mL (pulp)	(92)
*M. cavendish* green peel hydroaloholic extract	Human colorectal adenocarcinoma Caco-2 cells	┴Proliferation	29.7 ± 0.007% inhibition at 1000 μg/mL	(91)
Protocatechualdehyde	HCT116 and SW480	┴Proliferation; ↑apoptosis; ↓ (HDAC2)-initiated cyclin D1; ↓CDK4; ↓enzymatic activity of HDAC; ↓HDAC2; cell cycle arrest from G_1_ to S phase; ↑ATF3; ↑mRNA ATF3; ↑p-ERK1/2; ↑MAPK; ↑ PARP cleavage	71% inhibition at 200 μM and 58% inhibition at 200 μM 43% reduced cell viability at 200 μM and 56% reduced cell viability at 200 μM	(42, 98)
2-pentanone	HT29	┴ PGE_2_; ┴COX-2	400 μM	(59)
*Liver cancer*				
*Musa cavendish* green peel hydroaloholic extract	Hepatocellular carcinoma HepG2 cells	↑Cytotoxicity; ┴proliferation; ↑apoptosis; ↑necrosis; ↓MMP; ↑ROS	100 μg/mL; 100-400 μg/mL (apoptosis); 100, 200 and 400 μg/mL	(91)
Crude extracts (BPS and BR) of chloroform and acetone	HepG2 cells	↑Cytotoxicity	25 μg/mL (chloroform); 300 μg/mL (acetone)	(99)
4-epicyclomusalenone	HepG2 cells	↑Cytotoxicity	108 ± 1.8 μg/mL	
Cycloeucalenol acetate	HepG2 cells	↑Cytotoxicity	93 ± 1.5 μg/mL	
Chlorogenic acid	HepG2 cells	↑Cytotoxicity	382 ± 3.6μg/mL	
*Oral cancer*				
Ethyl acetate fraction of ethanol extract of banana soft piths (BSPs)	Human oral squamous cell carcinoma (OSCC) cell lines (HSC-4)	↑Cytotoxicity	26.95 μg/mL	(100)
*Prostate cancer*				
Aqueous banana flower extract	Epithelial cell line BPH-1 cells	┴Proliferation; cell cycle arrest at G_1_ phase; ↓cyclin D1; ↓cyclin dependent kinase (Cdk) 6; ↑p53; ↑p27; ┴ PGE_2_; ┴COX-2	2 mg/mL	(101)
Banana peel methanolic extract	LNCaP human prostate cancer cell line	┴Testosterone induced cell growth; ┴5*α*-reductase activity	25 μg/mL	(102)
*Skin cancer*				
*Musa cavendish* green peel hydroaloholic extract	Malignant melanoma A-375 cells	┴Proliferation; ↓MMP	100 μg/mL	(91)
Sucrier banana peel methanolic extracts	B16F10 mouse melanoma cells	↓MITF; ↑p-p38; ↑MITF protein degradation	100-500 μg/mL	(103)

Various symbols (↑, ↓ and ┴) indicate increase, decrease and inhibition in the obtained variables, respectively.

**Table 3:** *In vivo* anticancer activities of *Musa* spp. extracts.

Materials tested	Animal models	Effects and mechanisms	Dose	References
*Breast cancer*				
Aqueous extract of ripe banana	Erhlich’s ascites carcinoma cells in Swiss albino mice	Prolonged survival and reduced tumor development	2 g banana/day/mouse	(104)
MAL from the phloem exudates of *M. acuminate* pseudostem	Erhlich’s ascites carcinoma cells in Swiss albino mice	┴Tumor development; ↓neoangiogenesis; ↑survival	10mg/kg	(94)
*Colon cancer*				
Green banana flour (10%) as supplement	DMH-induced colon carcinogenesis in male Swiss mice	↓Number of aberrant crypt foci	Not specified	(105)

Various symbols (↑, ↓ and ┴) indicate increase, decrease and inhibition in the obtained variables, respectively.

#### Breast Cancer

Regarding *in vitro* studies, aqueous methanol extract of *Nendran* banana peel exhibited significant antitumor activity against the MCF-7 breast cancer cell line by inducing concentration-dependent apoptosis (89). Another *in vitro* experimental study showed that the anthocyanin extracted from methanol extracts of *M. acuminata* bract suppressed the proliferation of MCF-7 cells through induction of apoptosis (90). Additionally, *M. cavendish* green peel hydroalcoholic extract exhibited antiproliferative activity against the MCF-7 cell line at a concentration of 100 μg/mL (91). Additionally, hexane extract of banana (*M. sapientum*) peel and pulp was observed to be cytotoxic and blocked the proliferation of MCF-7 cells (92).

Aqueous fruit extract of ripe banana was utilized for evaluating its anticancer activity in Swiss albino mice bearing Erhlich ascites carcinoma cells. In comparison to the positive control, in which 100% of the animals died due to the carcinogenic effect, regular feeding of 2 g banana/day/mouse resulted in the growth suppression of malignant ascites leading to survival of 30% of the animals with Erhlich carcinoma which survived more than 35 days (104). Mannose-specific *M. acuminate* lectin (MAL) from the phloem exudates of *M. acuminate* pseudostem also demonstrated antitumor activity in Swiss albino mice bearing Erhlich carcinoma cells. Mechanistic studies showed initiation of apoptosis through the arrest of cell cycle progression at G2/M phase *via* stimulation of caspase-3, caspase-8 and caspase-9 with increased poly (ADP-ribose) polymerase (PARP) cleavage. It further triggered apoptosis through the phosphoinositide 3-kinase (PI3K)/Akt (also known as protein kinase B) signaling pathway, which inhibited the expression level of phosphorylated Akt (pAkt). MAL also blocked the phosphorylation of extracellular signal-regulated kinase 1 and 2 (ERK1/2) and c-Jun N-terminal kinase (JNK) (94).

#### Cervical Cancer

Ethanol extract of banana flower (*M. paradisiaca*) exhibited anticancer activity against HeLa cervical cancer cell line. The extract inhibited cellular proliferation and induced apoptosis, which was evident by the appearance of breaking up of the nuclei associated with significantly increased caspase-9 activity. The extract also blocked the cell cycle progression at the G_0_/G_1_ phase as well (93). Interestingly, the extract did not induce any toxicity on normal human peripheral lymphocytes. In a separate study, MAL exhibited similar cytotoxicity and antiproliferative activity against HeLa cells by initiating apoptosis and arresting cell cycle progression at the G2/M stage through activation of caspase-3, caspase-8 and caspase-9 associated with elevated PARP cleavage. It also induced apoptosis through the PI3K/Akt signaling pathway, in which it blocked the expression level of phosphorylated Akt (pAkt). It further inhibited the phosphorylation of ERK1/2 and JNK (94). Ferulic acid was isolated from banana peel using *Staphylococcus aureus*, and it exhibited antiproliferative and cytotoxic activities against HeLa cervical cancer cells by inducing DNA fragmentation (95). Additionally, the methanol extract of *M. acuminate* flower showed antiproliferative and cytotoxic potential against HeLa cells (96). An ethyl acetate fraction of *M. x paradisiaca* L. leaves also showed strong cytotoxic and anticancer activity against HeLa and A375 cervical cancer cell lines (97).

#### Colon Cancer

The hexane fraction of banana (*M. sapientum*) peel and pulp exhibited *in vitro* anticancer activity against HCT-116 colon carcinoma cell line. It was observed that the peel and pulp extract arrested cell growth by inducing cytotoxicity and blocked the proliferation of HCT-116 cells (92). In a separate study, *M. cavendish* green peel hydroalcoholic extract suppressed the proliferation of Caco-2 human colorectal adenocarcinoma cells (91). Protocatechualdehyde (PCA, 3,4-dihydroxybenzaldehyde), a polyphenol, was isolated from green cavendish bananas (106). It exhibited antiproliferative activity by triggering apoptosis in human colorectal carcinoma cells (HCT116 and SW480) in a concentration-dependent manner *via* histone deacetylase 2 (HDAC2)-initiated cyclin D1 suppression. It also downregulated the transcriptional level of the cyclin D1 gene and reduced the expression level of cyclin-dependent kinase 4 (CDK4). Additionally, PCA attenuated the enzymatic activity of HDAC and reduced the expression of HDAC2, but not HDAC1, thereby inducing cell cycle arrest at the G_1_ to S phase in both the cell lines tested (42). PCA increased the expression level of activating transcription factor 3 (ATF3) and ATF3-mediated apoptosis in human colorectal carcinoma (HCT116 and SW480 cell lines). PCA decreased the cell viability in a concentration-dependent manner by increasing the expression of ATF3 protein and mRNA ATF3 levels *via* phosphorylation of extracellular signal-regulated protein kinase 1 and 2 (ERK1/2) and p38 mitogen-activated protein kinase (MAPK) proteins and cleavage of PARP (98). Pettersson et al. (59) reported that 2-pentanone, a methyl propyl ketone present in banana, exhibited antiproliferative action by inhibiting the prostaglandin (PGE_2_) production and cyclooxygenase-2 (COX-2) protein expression in tumor necrosis factor-α (TNF-α)-stimulated colon cancer cells (HT29).

In an *in vivo* study, green banana flour (10%) was used as a dietary supplement to evaluate its anticancer potency in 1,2-dimethylhydrazine (DMH)-induced colon carcinogenesis in male Swiss mice over a 12-week experimental period. In all treated groups, it reduced the number of aberrant crypt foci (a colon cancer biomarker) in the colorectal mucosa, suggesting anticarcinogenic efficacy although the detail mechanism of action was not reported (105).

#### Liver Cancer

An anticancer study on *M. cavendish* green peel hydroalcoholic extract reported antiproliferative activity against HepG2 human hepatocellular carcinoma cell lines. The extract initiated both apoptosis and necrosis in a concentration-dependant manner, associated with alteration in cell morphology. It was also observed that it decreased the level of mitochondrial membrane potential (MMP) and increased reactive oxygen species (ROS) level (91). Another study has reported that various extracts of banana (*Musa* AAB var. Nanjanagudu Rasabale) pseudostem and rhizome demonstrated cytotoxicity against HepG2 cell lines. Crude chloroform and acetone extracts of the banana pseudostem and rhizome exhibited substantial cytotoxicity against the cell line tested (99). Bioactive compounds, such as 4-epicyclomusalenone and cycloeucalenol acetate, were isolated from chloroform extract, and chlorogenic acid was extracted and purified from the acetone extract of the banana rhizome. These compounds also exhibited potent cytotoxicity against HepG2 cell lines.

#### Oral Cancer

Ethyl acetate sub-fraction of the ethanol extract of banana (*M. paradisiaca*) soft piths (BSPs) exhibited potent cytotoxic and antiproliferative activity against the human oral squamous cell carcinoma (OSCC) cell line (HSC-4) (100).

#### Prostate Cancer

Aqueous banana flower extract exhibited anticancer activity against benign prostatic hyperplasia (BPH) *in vitro*. The banana flower extract at a concentration of 2 mg/mL reduced the viability of BPH-1 cells through cell-cycle arrest at the G_1_ phase. Moreover, it reduced the expression level of cyclin D1 and cyclin-dependentkinase6 (Cdk6) and elevated the expression level of p53 and p27 (101). It further reduced PGE_2_ production by inhibition of COX-2 enzymes during inflammation, which has shown to be the key factor in BPH-1 cell growth and proliferation. In another study, the methanol fraction of the banana peel inhibited testosterone-induced cell growth in a concentration-dependant manner against the androgen-responsive LNCaP human prostate carcinoma cell line by inhibiting 5*α*-reductase activity (102).

#### Skin Cancer

*M. cavendish* green peel hydroalcoholic extract demonstrated antiproliferative and cytotoxic activity against A-375 human malignant melanoma cells at a concentration of 100 μg/mL (91). Another study demonstrated that Sucrier banana peel methanolic extract induced inhibition of melanogenesis in B16F10 mouse melanoma cells by down-regulating microphthalmia-associated transcription factor (MITF) expression and p38 signaling pathway and up-regulating the phosphorylation of p38, which activated the MITF protein degradation at concentrations of 100-500 μg/mL (103).

### Clinical Studies

According to a hospital-based case-control analysis of Singapore Chinese esophageal cancer patients, esophageal cancer occurs at a higher incidence in male patients who eat fewer or no bananas in their diet and weekly consumption of banana reduces the risk of esophageal cancer (107). Moreover, based on a population-based case-control study, frequent consumption of bananas (8.9 g/day) lowers the risk of breast cancers (108). Clinical evidence from randomized controlled trials has been lacking. Hence, additional clinical trials are needed to understand the therapeutic effectiveness of banana constituents. Banana is a very acceptable food for all types of communities. So, banana should be a part of regular diet to reduce the incidence of esophageal cancer and breast cancer; however other similar clinical studies should be performed to analyze the therapeutic activity of banana on other cancer types.

## Conclusion and Future Perspectives

**Figure 7:**
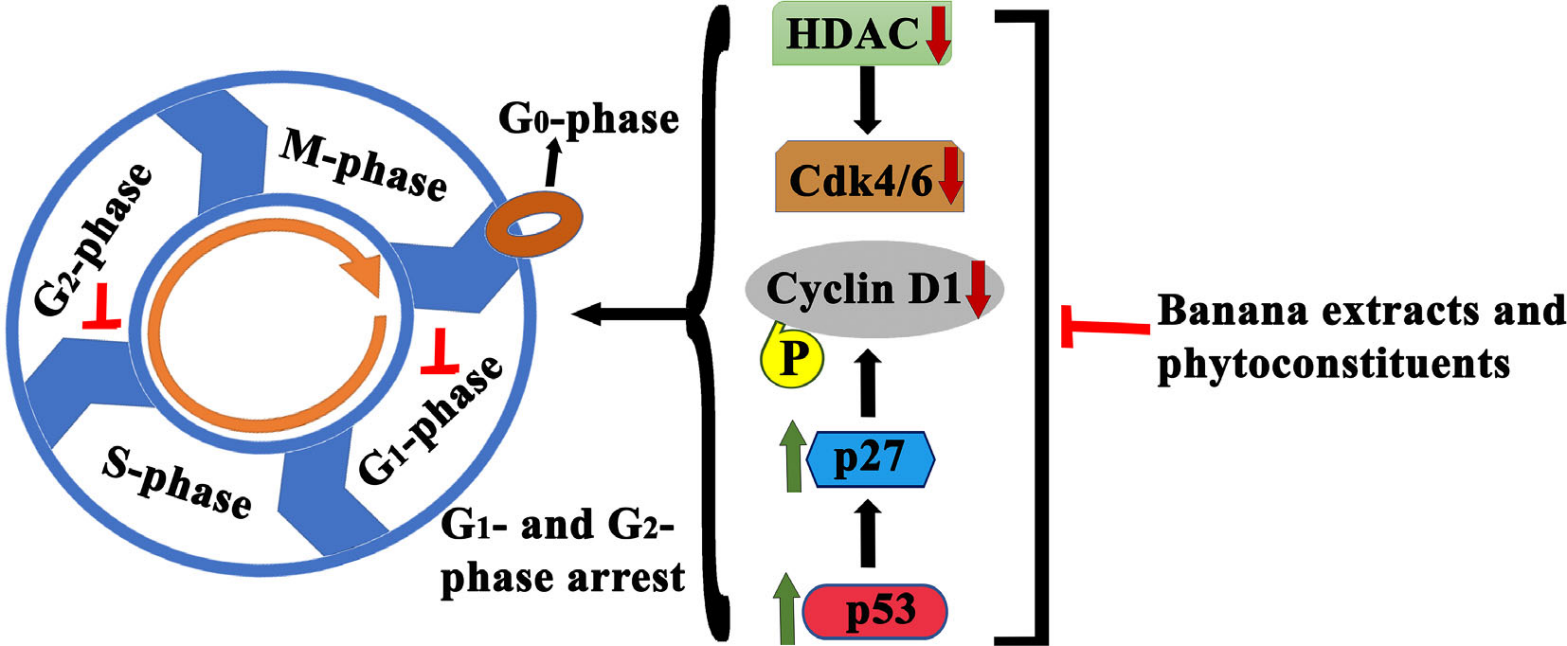
Schematic illustration of anticancer effects of banana extracts and its phytoconstituents through cell cycle arrest. Multiple studies found cell cycle arrest effects of banana extracts and its phytoconstituents at various check points which lead to the proliferation inhibition.

**Figure 8:**
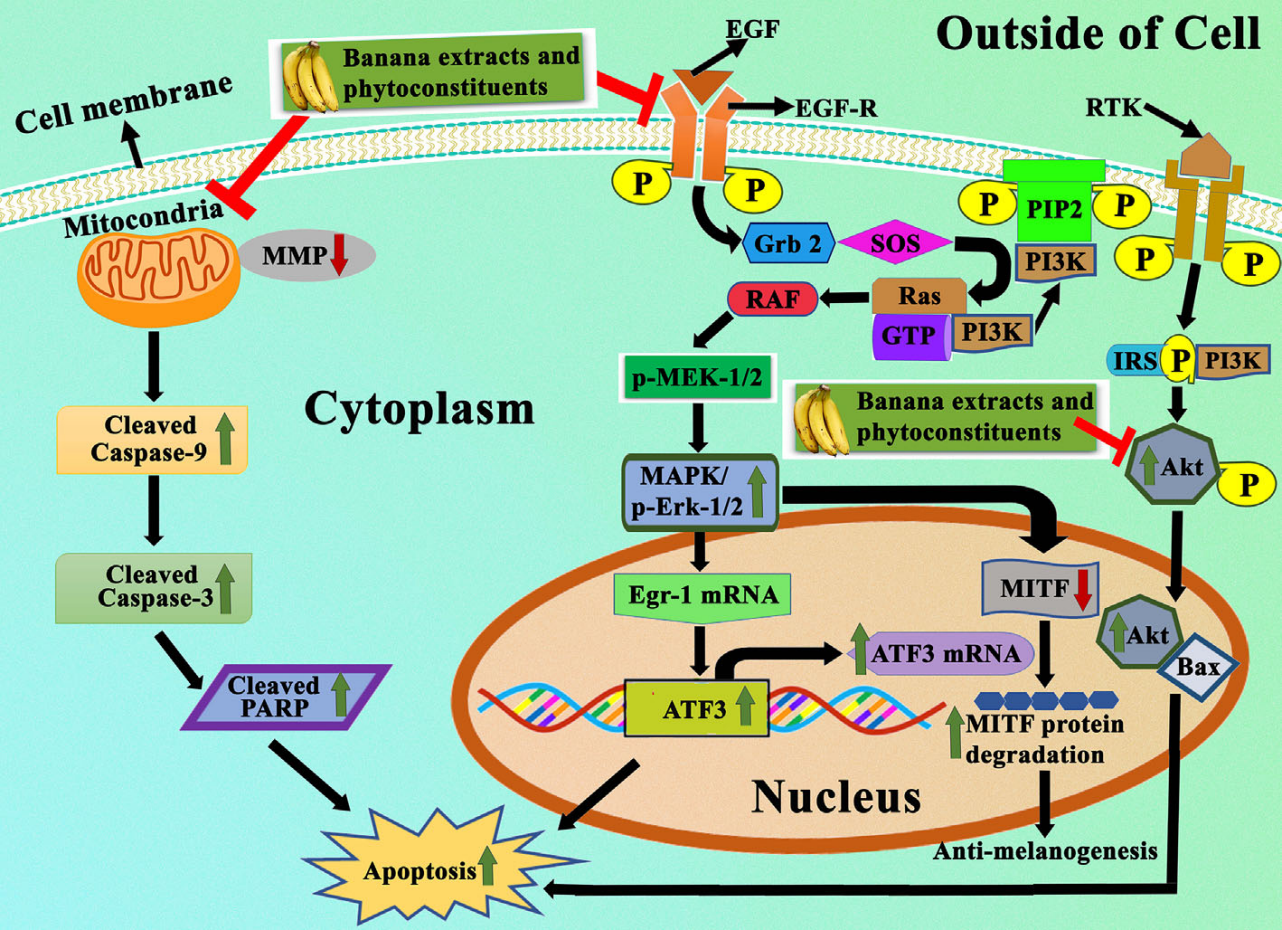
Representation of apoptotic effects of banana extracts and its bioactive phytoconstituents. Under the apoptotic effects, banana extracts and its bioactive phytoconstituent can induce the expression of Bax, caspase-3, caspase-9, cleaved PARP and block the expression of Bcl-2. It also regulates the MAPK/ERK1/2 signaling pathway, PI3K-Aktsignaling pathway, in which the expressional levels of p-ERK1/2, ATF3, and ATF3 are modulated.

**Figure 9:**
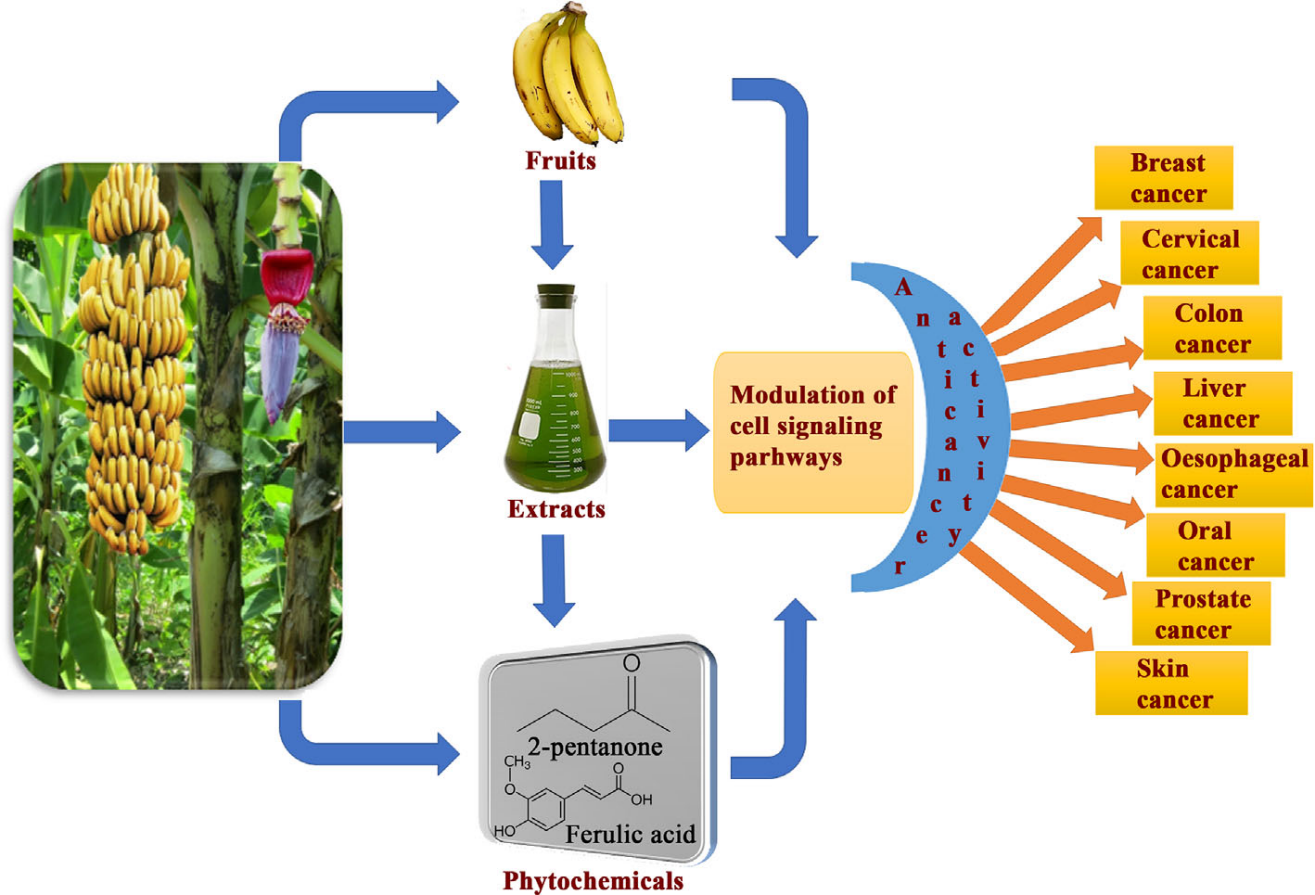
Overview of consumption banana fruits, extracts and its phytoconstituents exhibiting cancer preventive and anticancer activity against various cancer types through modulation of diverse cell signaling pathways.

Banana is a magnificent plant that has been cultivated for food and medicinal purposes for thousands of years. We have summarized various secondary metabolites from different banana plant belonging to *Musa* species in this review. In this article, we analyzed the *in vitro* and *in vivo* chemopreventive and chemotherapeutic effects of banana and its phytochemicals and also the toxicity of specific active components. The phytoconstituents were isolated from different varieties of banana belonging to *Musa* species, such as *M. accuminata, M. balbisiana*, and other varieties; among which majority of the phytoconstituents belonging to *M. accuminata* which exhibited chemopreventive and anticancer activities. There are variations in the phytochemical compositions of different varieties of banana due to soil, temperature, banana variety, maturation stage, processing location, and other variables. While the banana fruit (including the pulp and peel) has gained a lot of interest, other parts of the banana plant, such as the leaf, flower, and stem, have also been investigated for anticancer purposes. In addition to banana extracts and fractions, some phytoconstituents, including ferulic acid, protocatechualdehyde, 2-pentanone, 4-epicyclomusalenone, cycloeucalenol acetate, and chlorogenic acid, have been shown to exhibit cancer preventative and anticancer therapeutic abilities. Cancer preventive studies are limited, although two such study is reported where consumption of banana can reduce the incidence of esophageal cancer (107) and breast cancers (108); however other similar studies should be performed to analyze the cancer preventive activity of banana on other cancer types. We have also addressed the various mechanisms by which numerous extracts of banana and their active constituents carry out their biological functions in cancer. Bioactive components present in bananas have exhibited momentous cancer preventive and anticancer activities utilizing various mechanisms, which include cytotoxicity, cell cycle arrest, apoptosis of cancer cells, antioxidant, and anti-inflammatory effects. The cell cycle is a sequence of events in a cell that split it into two cells. Cell cycle check points are control mechanisms that ensure the proper progression. Banana and its phytoconstituents induced cell cycle arrest at these check points to halt the progression through the cell cycle of neoplastic cells (**Figure 7**). Banana phytoconstituents also demonstrate various mechanisms in the modulation of diverse, dysregulated signaling pathways in order to prohibit cancer progression (**Figure 8**). Banana and its phytochemicals are able to induce changes in expression level of some commonly known genes to regulate well-known signaling networks, such as MAPK signaling pathways, the ERK signaling pathway, the ERK1/2 signaling pathway, and HDAC2 signaling pathway, in addition to inhibiting pro-inflammatory mediators, such as COX-2. By modulating these pathways, phytoconstituents from banana restraint cell proliferation, adhesion, invasion, and angiogenesis in breast, cervical, colorectal, esophageal, hepatic, oral, prostate, and skin cancers (**Figure 9**).

Poor bioavailability and bioaccessibility of various phytochemical constituents of banana are barriers to their therapeutic use. The weak bioavailability as well as bioaccessibility were due to the initial hepatic first pass effect, poor absorption of the intestines, and low solubility (Sidhu and Zafar, 2018). Different aspects, such as banana processing and variety, often affect bioavailability and accessibility.

Despite the vast amount of research that has been performed and documented over the last few decades, the bulk of the findings cited in this review are focused on *in vitro* experiments. Breast, cervical, colorectal, esophageal, hepatic, oral, prostate, and skin cancers are only a few of the cancer types affected by banana and its phytochemicals. The reviewed literature reveals the promise that banana and its phytochemicals can be used in chemotherapy for different forms of cancer. The role of banana and its phytoconstituents on breast cancer and colon cancer have been studied extensively in both *in vitro* and *in vivo* research. Findings from *in vivo* studies on other cancer types and clinical situations are sparse. With encouraging preclinical data, mechanistic investigations on anticancer actions of the components of banana are warranted. The translation impact of available research findings is restricted by the lack of well-designed, prospective clinical studies and safety evaluation of banana extracts and constituents in humans. The potential pharmacokinetic constraints of banana phytochemicals highlight the need to establish efficient and well-regulated delivery mechanisms for optimized delivery systems against various malignancies. More experiments on novel molecular targets and signaling pathways of banana bioactive materials, in addition to well-controlled clinical trials, will increase the therapeutic potential of this popular and medicinal fruit for cancer prevention and treatment. According to the selective toxicity tests, bananas and their main components are safe. However, more research is to be carried out to see whether the same favourable safety profile occurs in human subjects, and to determine which banana secondary metabolites may be cytotoxic, if any. Moreover, banana-derived products may be utilized as an adjuvant to various chemotherapeutic drugs (which have many adverse side effects) for a variety of cancer subtypes.

Our systematic study and review of limitations also identify various future research paths. Although numerous bioactive banana compounds have been identified, further research into the anticancer ability of these phytochemicals found in bananas is required. Furthermore, since the bulk of research is limited to *in vitro* studies, more *in vivo* mechanistic experiments should be performed. Provided the positive anticancer findings presented in this study, randomized clinical trials involving banana phytochemicals should be carried out. However, further research into the anticancer ability of other important phytochemicals found in bananas is warranted. Characterization of different phytochemicals found in bananas that function alone or synergistically with other compounds or established drugs to have cancer ameliorative or protective effects is also required. In conclusion, based on our in-depth analysis of the existing literature, banana extracts and the isolated phytoconstituents found in banana present as promising medicinal agents for cancer prevention and these agents could also be developed as multi-targeted drugs for cancer pharmacotherapy.

## Data Availability Statement

The original contributions presented in the study are included in the article/supplementary material. Further inquiries can be directed to the corresponding authors.

## Author Contributions

Conceptualization: AM. Literature search and collection: SBo, SBa, and PD. Writing—original draft preparation: AM, SBa, and ES. Writing—review and editing: AM, AA, and AB. Supervision: AM. Project administration: AB. All authors contributed to the article and approved the submitted version.

## Conflict of Interest

The authors declare that the research was conducted in the absence of any commercial or financial relationships that could be construed as a potential conflict of interest.
